# Dietary inflammatory index, risk of incident hypertension, and effect modification from BMI

**DOI:** 10.1186/s12937-020-00577-1

**Published:** 2020-06-25

**Authors:** Conor-James MacDonald, Nasser Laouali, Anne-Laure Madika, Francesca Romana Mancini, Marie-Christine Boutron-Ruault

**Affiliations:** 1 INSERM (Institut National de la Santé et de la Recherche Médicale) U1018, Center for Research in Epidemiology and Population Health (CESP), Institut Gustave Roussy, Villejuif, France; 2 Université Paris-Saclay, Université Paris-Sud, Villejuif, France; 3 Université de Lille, CHU Lille, EA 2694 - Santé publique : épidémiologie et qualité des soins, F-59000 Lille, France

**Keywords:** Inflammation, Dietary inflammatory index, Hypertension, Nutrition, Epidemiology

## Abstract

**Introduction:**

Previous studies have identified a positive association between the inflammatory potential of the diet and hypertension. It is not known if BMI is an effect modifier for this association, nor if the association is dose-respondent. This study aimed to assess the association between the dietary inflammatory index (DII) and the risk of hypertension, and assess any effect modification from BMI.

**Methods:**

Data from the E3N cohort study, a French prospective population-based study initiated in 1990 was used. From the women in the study, we included those who completed a detailed diet history questionnaire, and who did not have prevalent hypertension or cardiovascular disease at baseline, resulting in 46,652 women. The adapted DII was assessed with data from the dietary questionnaire. Hypertension cases were self-reported and verified through a drug-reimbursement database. Cox proportional hazard models were used to calculate hazard ratios. Spline regression was used to determine any dose-respondent relationship.

**Results:**

During 884,267 person-years, 13,183 cases of incident hypertension were identified. The median DII in the population was slightly pro-inflammatory (DII = + 0.44). A highly pro-inflammatory diet (DII >  3.0) was associated with a slight increase in hypertension risk (HR_Q1-Q5_ = 1.07 [1.02, 1.13]). Evidence was observed for effect modification from BMI, with associations strongest amongst women in the 18.5–21.0 BMI range (HR_Q1-Q5_ = 1.17 [1.06, 1.29]). A weak dose-respondent relationship was observed.

**Conclusion:**

Evidence for a weak association between DII and hypertension was observed. Associations were stronger amongst healthy-lean women.

## Introduction

Hypertension is a major risk-factor for cardiovascular diseases (CVD), and is well known to be associated with chronic inflammation [1–3]. Chronic inflammation is a consistently documented biological feature of aging, with biomarkers of inflammation such as C-reactive protein (CRP), interleukin-1B/6, and tumour necrosis factor alpha increasing with age, even in the absence of infection [4]. Inflammation is increased in obesity, which is a major risk factor for CVD [5]. It is not clear whether inflammation is a cause, or an effect of hypertension; inflammatory markers can be elevated in cases of hypertension [6, 7], and multiple prospective trials have also linked increased inflammation to higher risks of incident hypertension [8–11].

Many environmental and lifestyle factors are associated with inflammation, and one modifiable lifestyle factor of particular interest is the diet. It has been demonstrated that certain inflammatory markers increase after consuming saturated fatty acids and decrease after ingesting certain unsaturated fatty acids [12, 13]. In addition, some dietary patterns are linked to inflammation. The DASH diet [14], which is high in dietary fibre and low in fat, has been shown to decrease levels of inflammatory markers [15], and is associated with reduced blood pressure [14]. Similarly, the Mediterranean diet pattern, which is high in fruit and vegetables, and healthy fats, has been associated with reduced concentrations of CRP [16], and has been associated with reduced risk for many non-communicable diseases (NCDs). Inversely, the Western diet, characterised by intakes of processed meat, and refined carbohydrates, has been implicated with increases in inflammatory markers [17] and appears to be a risk factor for several NCDs.

Recently, the dietary inflammatory index (DII) [18, 19] has been developed to reflect inflammatory potential of the diet and scores an individual’s diet on a continuum from anti to pro-inflammatory [20]. The DII has been shown to be associated with diseases such as diabetes [21], obesity [22], asthma [23], cancer [24, 25], myocardial infarction, stroke and CVD mortality [26–28]. Some prior studies have prospectively assessed the inflammatory potential of the diet in relation to hypertension or blood pressure [29, 30], both finding a positive association between the DII and hypertension diagnosis, or blood pressure increases, but did not report the shape of the association. Excess body weight is a major risk factor for hypertension, and it is associated with low rate chronic inflammation [31], however, it is still unknown whether BMI is an effect modifier for the reported association between DII and hypertension.

The aim of the current study was to determine if an a priori pro-inflammatory diet assessed using DII was associated with an increased risk of hypertension in a large French cohort, and to determine if BMI acted as an effect modifier for any associations observed.

## Methods

### Study population

The Etude Epidémiologique de femmes de la Mutuelle Générale de l’Education (E3N) [32] is a French prospective cohort started in 1990 comprising 98,995 women aged 40–65 years at baseline and insured by the MGEN (Mutuelle Générale de l’Education Nationale), a health insurance plan for workers in the National Education System and their families. The objective of E3N was to study the main risk factors of cancer and chronic diseases. The E3N is the French component of the European Prospective Investigation into Cancer and Nutrition. The cohort received ethical approval from the French National Commission for Computerized Data and Individual Freedom (Commission Nationale Informatique et Libertés), and all participants in the study signed an informed consent.

Participants returned mailed questionnaires on lifestyle information and disease occurrence every 2 to 3 years. The average response rate at each questionnaire cycle was 83%, and the total loss to follow-up was 3%.

From the 74,522 women who responded to a dietary questionnaire in 1993 we excluded those women with prevalent hypertension, coronary disease or stroke (*n* = 26,974) before or at the 1993 questionnaire, and those with unrealistic energy consumption (the 1st and 99th percentiles of the distribution of the ratio of energy intake to the basal metabolic rate computed on the basis of age, height, and weight, *n* = 896) [33]. The final study population included 46,652 women.

### Assessment of the dietary inflammatory index

In 1993 dietary data was collected using a two-part questionnaire detailing consumption of 208 food items during the year prior to the questionnaire, which has been shown to be reproducible and valid to classify study subjects according to their food and nutrient intake over a one-year period [34]. Women were asked to answer questions about quantities and frequencies of consumption of food groups. Eleven possible responses were available, never or less than once a month; 1 to 3 times a month, and 1 to 7 times a week. A photo booklet was added to help estimate portion sizes [35]. From this questionnaire and using a detailed food composition table, mean daily intakes of energy (excluding energy from alcohol), alcohol, and nutrients were estimated.

The adapted DII was estimated as previously described [21]. Briefly, the adapted dietary inflammatory index proposed by Woudenberg et al [19] was used in combination with the updated dietary components weights by Shivappa et al [36] instead of the weights proposed by Cavicchia et al [37]. This DII has been proposed on the basis of nutritional rationale. First, the inflammatory weights of dietary components are multiplied by the standardised energy adjusted intake, which acts to reduce between-person variation. Second, the intake of all components are standardised by subtracting the mean intake of the population (in this case E3N, *n* = 74,522) from the individual’s intake, and then divided by the standard deviation of the intake from the population. Finally, the inflammatory effects of energy and total fat were not calculated separately, as they were considered to be equivalent to the sum of the inflammatory effects of all energy-providing macronutrients, and all separate fatty acids, respectively. Similarly, as ethanol was used in the estimation of the DII, we did not consider separately the inflammatory effect from specific alcoholic beverages.

A total of 32 of the 35 possible dietary components were used for DII calculation (see Supplementary Table 1) based on the food frequency questionnaire. A positive DII score is representative of a pro-inflammatory diet, and negative values of an anti-inflammatory diet.

### Hypertension assessment

Participants were asked to report whether they had hypertension at baseline (1993) and in each follow-up questionnaire (1994, 1997, 2000, 2002, 2005, 2008, 2011, and 2014), the date of diagnosis, and the use of antihypertensive treatments. The month and year of diagnosis were provided for most cases (69%). For individuals who were missing the month of diagnosis (14% of cases), it was imputed to June of the year of diagnosis. The median time between the date of diagnosis and the date of response to the first questionnaire after diagnosis was 12 months. Thus, for the cases with no year of diagnosis (*n* = 17%), we assigned it to be 12 months before they reported hypertension in a questionnaire. In 2004, a drug reimbursement database became available for 97.6% of participants. We used the self-reported date of diagnosis or the first date of drug reimbursement for antihypertensive medications (Anatomical Therapeutic Chemical Classification System codes C02, C03, C07, C08, and C09) whatever happened first, as the date of diagnosis for cases identified after 2004.

In addition, using the information of the MGEN health insurance plan drug claim database, we assessed the validity of self-reported hypertension within the E3N cohort. We compared hypertension self-report to antihypertensive drug reimbursement (any of the above specified codes). A positive predictive value of 82% was observed [38].

### Assessment of covariates

Family history of hypertension, education (no high school diploma, high school diploma), and smoking (ever smoker, current smoker, or never smoker) were based on self-reports, and for diabetes and treated dyslipidaemia we used cases which had been validated through the use of a drug reimbursement database [39]. A Mediterranean/prudent diet score was determined from dietary data using principal component analysis, as previously described [40].

We assessed usual physical activity with a questionnaire in 1993 that included questions on weekly hours spent walking, cycling and performing light and heavy household chores, and questions on recreational activities and sports (e.g., swimming and tennis) considering the winter and summer seasons. It included questions on the time spent walking (to work, shopping, and leisure time), cycling (to work, shopping, and leisure time), housework, and sports activities (such as racket sports, swimming). Metabolic equivalents (METs) per week were estimated by multiplying the hourly average METs for each item based on values from the Compendium of Physical Activities [41] by the reported activity duration.

Self-reported height and weight at baseline were used to calculate body mass index (BMI), defined as weight (kg) divided by squared height (m^2^). In the cohort, self-reported anthropometry is considered reliable from a validation study [42].

### Statistical analysis

Participants were split into quintiles depending on DII score depending on the population distribution. Characteristics between participants were tabulated (see Table 1) depending on their quintile of DII. Correlations between variables were assessed using Pearson’s R.

**Table 1:** Participant demographics depending on population distributions of adapted DII score

Variables (mean, SD)	Q1 (< − 2.7) (***n*** = 9331)	Q2 (− 2.7 - -0.4) (***n*** = 9330)	Q3 (− 0.4–1.3) (***n*** = 9330)	Q4 (1.3–3.0) (***n*** = 9330)	Q5 (> 3.0) (***n*** = 9331)
DII	− 5.3 (2.5)	− 1.5 (0.6)	0.4 (0.5)	2.1 (0.5)	4.6 (1.3)
Age (years)	50.6 (6.2)	50.5 (6.3)	50.4 (6.3)	49.9 (6.2)	49.4 (6.2)
BMI (kg / m^2^)	22.9 (3.0)	22.5 (2.8)	22.2 (2.7)	22.0 (2.7)	21.8 (2.7)
Total physical activity (MET-hours/ week)	57.4 (31.1)	55.7 (30.6)	54.0 (29.3)	53.1 (29.0)	51.2 (28.7)
Diabetes (%)	0.9	0.5	0.6	0.3	0.2
Dyslipidaemia (%)	6.0	6.5	5.4	4.8	3.7
Family history CVD (%)	33.9	33.7	33.9	33.8	33.4
Prior cancer (%)	6.5	6.9	6.9	6.5	7.1
Education (> high school) (%)	87.0	86.5	86.5	86.6	86.0
Smoking (Never/X/current) (n)	4519/3421/1409	4756/3302/1290	4933/3172/1243	4994/2986/1368	5089/2903/1356
**Dietary variables (median, SD)**					
Total energy (Kcal)	2118.0 (539.6)	2027.7 (517.0)	2031.8 (522.6)	2038.0 (515.1)	2174.4 (582.4)
Salt (mg / day)	2821.0 (939.0)	2665.2 (862.9)	2674.7 (863.5)	2670.8 (866.2)	2846.4 (957.7)
Potassium (mg / day)	4489.0 (1120.4)	3908.9 (897.1)	3643.6 (884.0)	3424.9 (819.5)	3225.0 (847.0)
Vegetables (g / day)	257.1 (118.6)	185.7 (5.8)	142.9 (74.2)	114.3 (66.1)	85.7 (58.5)
Fruit (g / day)	323.4 (211.2)	262.6 (161.7)	231.0 (141.8)	189.3 (124.4)	140.6 (122.1)
Dietary fibre (g / day)	31.1 (8.3)	25.7 (6.3)	23.4 (6.1)	21.5 (5.9)	19.8 (6.1)
Processed meat (g / day)	14.5 (19.9)	15.1 (17.6)	15.7 (16.5)	16.4 (16.7)	17.1 (17.7)
Alcohol (g / day)	6.3 (12.7)	6.9 (13.1)	7.2 (13.1)	7.2 (13.8)	7.3 (15.6)
Total fats (g / day)	87.6 (26.2)	83.5 (25.2)	83.2 (25.6)	84.0 (25.5)	91.4 (29.8)
Mediterranean diet score	1.0 (0.9)	0.3 (0.6)	−0.2 (0.6)	−0.5 (0.5)	−1.0 (0.6)

Hazards ratios and 95% confidence intervals were estimated from Cox regression models with age as the time scale. Time at entry was the age at the beginning of follow-up (1993), exit time was the age when participants were diagnosed with hypertension, died (dates of death were obtained from the participants’ medical insurance records), were lost to follow-up, or were censored at the end of the follow-up period (June 15, 2014), whichever occurred first. *P*-values for trends were calculated using the median category value as a semi-continuous variable in the models.

Four models assessing DII as the exposure were assessed; the first was controlled for age as the timeline (Model 0). Multivariable models were first adjusted for various known risk-factors for hypertension: total physical activity (MET-hours/week, continuous), family history of cardiovascular disease (yes/no), smoking (never, former, and current at baseline), education (no high school diploma, high school diploma) (Model 1), diabetes and dyslipidaemia status (Model 2), and finally BMI (kg/m^2^, continuous) (Model 3). As dyslipidaemia and diabetes may affect both exposure and outcome, models with and without these variables were assessed, but no difference in estimates was obtained, thus the variables were retained.

Spline regression with 5 degrees of freedom was used to assess the dose-response relationship between DII and the risk of incident hypertension. Tests for interactions (ANOVA) were performed for BMI as a continuous variable to determine if it was an effect modifier. As a hypothesis generating exercise, this was also done for smoking status (never, former, and current at baseline), and Mediterranean diet score (continuous). If tests were indicative of effect modification, models were stratified on this variable.

Missing values (less than 5% of participants) were imputed using the mean for continuous, or median for categorical variables. All statistical analyses used R version 3.5.1 (www.r-project.org) and the survival package (www.github.com/therneau/survival), with an alpha of statistical significance equal to 0.05. Results from Cox-models were interpreted as hazard ratios (HR) (95% confidence interval (CI)). The proportional hazards assumption was assessed by plotting the Schoenfeld residuals using the cox.zph package in R.

### Sensitivity analysis

Several sensitivity analyses were performed. A model with DII categorised dichotomously as negative (anti-inflammatory) or positive (pro-inflammatory) was assessed, in the same method as previously described. We also calculated the DII using only the women in the final study cohort as reference (*n* = 46,652) when the mean intake of the population was subtracted, as opposed to the 74,522 women of the considered DII score. Similarly, an alternative DII score which takes into account the inflammatory potential from total energy intake was assessed in a sensitivity analysis (“non-adapted” DII, method of Shivappa et al [36]). Results for these scores are presented in the Supplementary data. In order to account for reverse causation, we excluded cases diagnosed within 5 and then within 10 years.

## Results

During 884,267 person-years, 13,183 cases of incident hypertension were identified. The mean (s.d.) age of women in this study was 50.1 (6.3), and the mean BMI was 22.3 (2.8). The mean DII score was + 0.44 (3.6) with a minimum of − 24.8 and a maximum of 12.2. Women with a higher DII score were younger, less active, consumed fewer vegetables and fruits, and less fibre, and were less likely to have obesity, diabetes, or dyslipidaemia (Table 1). DII showed high negative correlation with intakes of vegetables (*r* = − 0.61), fibre (*r* = − 0.57), fresh fruit (*r* = − 0.43), and was even more strongly negatively correlated with the Mediterranean diet score (*r* = − 0.79). Weaker positive correlations were observed between DII and added sugars (*r* = 0.25), white bread (*r* = 0.24), dairy-based desserts (*r* = 0.17), cheese (*r* = 0.17), fried potatoes (*r* = 0.16), fast foods such as burgers (*r* = 0.14), pâté (*r* = 0.11), and sausages (*r* = 0.10).

**Fig. 1:**
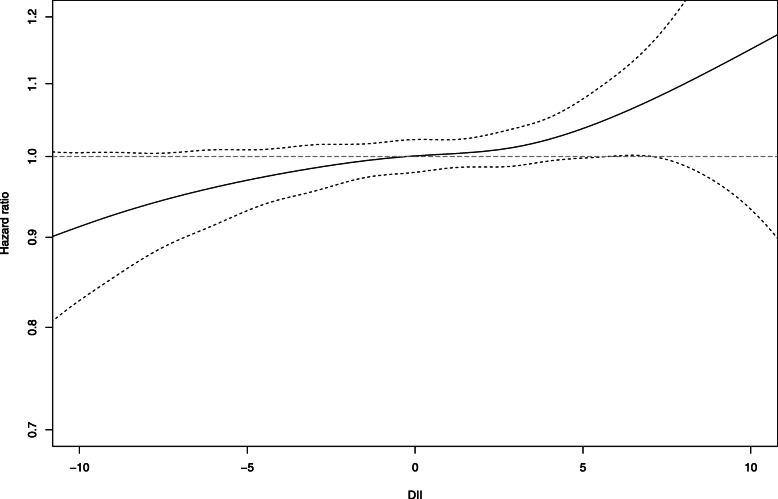
Spline regression showing relationship between adapted DII and hazard ratio for hypertension. Dashed line indicates 95% confidence interval. *P*-linear = 0.003, *P*-non-linear = 0.82

Increasing DII score was associated with an increased risk of hypertension after adjustment for BMI (HR_Q1-Q5_ = 1.07 [1.02: 1.13], p for trend = 0.003) (Table 2). Using spline regression, evidence was observed for a dose respondent relationship, with a decreased risk of hypertension corresponding to negative values of the DII (Fig. 1). Considering the positive/negative classification of the DII, a positive score (pro-inflammatory) was associated with a small borderline significant increase in risk for incident hypertension, only after adjustment for BMI (HR_+_ = 1.03 [0.99: 1.06], *p* = 0.10). The proportional hazards assumption was verified graphically.

**Table 2:** BMI adjusted and multivariate adjusted cox proportional hazard models for incident hypertension risk based on distributions of adapted dietary inflammatory index

Quintiles of DII	Q1 (< − 2.7) (***n*** = 9331)	Q2 (− 2.7 - -0.4) (***n*** = 9330)	Q3 (− 0.4–1.3) (***n*** = 9330)	Q4 (1.3–3.0) (***n*** = 9330)	Q5 (> 3.0) (***n*** = 9331)	***P*** for trend
Cases	2697	2699	2664	2555	2568	
Person years	175,873	176,292	176,738	177,652	177,712	
*M0*	*ref*	1.00 [0.95: 1.06]	0.99 [0.94: 1.04]	0.95 [0.90: 1.01]	0.97 [0.92: 1.03]	0.11
*M1*	*ref*	1.00 [0.94: 1.05]	0.98 [0.93: 1.03]	0.94 [0.89: 0.99]	0.96 [0.91: 1.01]	**0.02**
*M2*	*ref*	1.00 [0.94: 1.05]	0.98 [0.93: 1.04]	0.95 [0.89: 1.00]	0.97 [0.91: 1.03]	0.10
*M3*	*ref*	1.03 [0.98: 1.09]	1.04 [0.99: 1.10]	1.03 [0.97: 1.08]	1.07 [1.01: 1.13]	**0.003**

M0 with age as timescale; M1 adjusted for physical activity, smoking, family history of CVD, and education level; M2 M1 + diabetes and dyslipidaemia at baseline; M3 M2 + BMI

Evidence for interaction between BMI and DII on incidence of hypertension was observed (p for interaction = 0.0002). Models were stratified on BMI, in the following groups: BMI < 18.49, 18.50–21.49, 21.50–24.99, > 25.00 which were chosen due to the large number of women in the healthy range. Associations were only observed in participants with a BMI in the 18.50–21.49 range (HR_Q1-Q5_ = 1.17 [1.06: 1.29], *p* for trend = 0.002) (Table 3), and were not present in high-normal BMI (21.50–24.99) (HR_Q1-Q5_ = 1.05 [0.97: 1.14], p for trend = 0.22), overweight (> 25.00) (HR_Q1-Q5_ = 0.92 [0.86: 1.04], p for trend = 0.15) or underweight (< 18.50) (HR_Q1-Q5_ = 1.06 [0.79: 1.43], *p* for trend = 0.99) individuals (Table 3).

**Table 3:** Model stratified on BMI. Adjusted for physical activity, smoking, family history of CVD, education level, dyslipidaemia and diabetes

	BMI ***(Kg / m***^***2***^***)*** <= 18.49 (***n*** = 2141)	BMI ***(Kg / m***^***2***^***)*** 18.50–21.49 (***n*** = 18,566)	BMI ***(Kg / m***^***2***^***)*** 21.50–24.99 (***n*** = 19,370)	BMI ***(Kg / m***^***2***^***)*** > 25.00 (***n*** = 6526)
	HR [95% CI]	HR [95% CI]	HR [95% CI]	HR [95% CI]
**Quintiles of DII**				
**Q1 (< − 2.7)**	*ref*	*ref*	*ref*	*ref*
**Q2 (−2.7 – −0.4)**	1.14 [0.81: 1.59]	1.03 [0.93: 1.15]	1.04 [0.96: 1.12]	1.04 [0.93: 1.15]
**Q3 (−0.4–1.3)**	1.01 [0.74: 1.40]	1.10 [1.00: 1.22]	1.06 [0.98: 1.14]	0.96 [0.86: 1.08]
**Q4 (1.3–3.0)**	0.97 [0.71: 1.33]	1.05 [0.95: 1.16]	1.03 [0.96: 1.11]	0.97 [0.86: 1.09]
**Q5 (> 3.0)**	1.06 [0.79: 1.43]	1.17 [1.06: 1.29]	1.05 [0.97: 1.14]	0.92 [0.86: 1.04]
***p*-trend**	0.99	**0.002**	0.22	0.15

In sensitivity analysis excluding participants diagnosed within 5 and 10 years, results were similar (data not tabulated). Results were unchanged when assessing the calibrated DII score, which showed near perfect correlation with the score calculated for the whole population (*r* = 0.999). Similarly, results were unchanged when using the “non-adapted” DII as calculated by Shivappa et al (Supplementary Tables 2 and 3).

## Discussion

The results from this large prospective study suggest that a pro-inflammatory diet is weakly associated with an increased risk of hypertension, compared to an anti-inflammatory diet. These results were independent of known risk factors for hypertension onset such as BMI, physical activity, and smoking, and co-morbidities such as dyslipidaemia and diabetes. Evidence for effect modification from BMI was observed, with associations strongest amongst women in the 18.50–21.49 range.

In this cohort, a pro-inflammatory diet was associated with increased consumption of processed meats, total fat, added sugars, and alcohol. Women with a higher DII were typically younger, less often obese, and less likely to have prevalent diabetes or dyslipidaemia. A negative DII, indicating an anti-inflammatory diet was associated with increased consumption of fruit and vegetables, dietary fibre, a higher level of education, and higher levels of physical activity. These aspects are similar to other dietary indexes which have been associated with the risk of cardiovascular diseases and hypertension, such as the Mediterranean diet [43], which is characterised by high fruit, vegetable, and grain intake, or inversely with the Western diet score which is characterised by high fat and processed food intakes.

A number of smaller prospective studies have assessed DII and the risk of hypertension or changes in blood-pressure. In the French SUVIMAX study which included both men and women [30], a pro-inflammatory diet at baseline was associated with small increases in blood-pressure and incident metabolic syndrome. Similarly, in The Australian Longitudinal Study on Women’s Health (ALSWH) [29], a pro-inflammatory diet was associated with an odds ratio of 1.24 for incident hypertension, (comparisons were made between a positive and negative DII score). In the ALSWH, the mean BMI at baseline was higher, meaning that the populations are not entirely comparable. In both of these studies, the baseline DII was slightly higher, and both models adjusted for energy intake, as they both used a DII which did not take into account the effect from total calorific intake. In this study, we assessed a similar version of the score in a sensitivity analysis, and observed no major difference in our reported HRs.

Associations between DII and hypertension were not apparent until modelling was controlled for BMI, which is likely due to a tendency for DII to be associated with a slightly lower weight in this population, thus BMI represented a significant negative confounder. Effect modification was observed from BMI, with only women in the 18.50–21.49 range showing positive associations between DII and hypertension. This is possibly due to the low-risk profile, and low levels of chronic inflammation associated with women in this weight range making them more susceptible to dietary induced inflammation [20]. Persons with excess weight or obesity have increased levels of inflammation [31], and any increase induced from the diet over a certain level of inflammation may have little to no effect. Indeed previous research has found that amongst normal weight individuals, circulating CRP is around 1.2 mg/L, compared to 1.9 mg/L in overweight individuals [44]. In a study comparing hs-CRP between normal-weight participants with high and low DII scores, those with a low score had a mean CRP of 0.93 mg/L, compared to 1.02 mg/L in those with higher DII [20], although the odds of having CRP >  3 mg/L increased with higher DII.

The DII score was highly inversely associated with the Mediterranean diet score and intakes of fibre. The Mediterranean diet is characterised by anti-inflammatory properties [16], thus it is not unexpected that there was a correlation with DII. It is likely that the beneficial associations reported from adherence to the Mediterranean diet are mediated in part through anti-inflammatory effects, as is observed through the DII. However, due to the lifestyle factors typically associated with the Mediterranean diet such as conviviality, regular physical activity and adequate rest [45], it is perhaps not the whole picture.

This study has a number of strengths, primarily its size which includes a large number of cases, the long duration of follow up, and low loss to follow up. We were able to control for known risk factors for hypertension including BMI, physical activity, smoking, family history of CVD, and co-morbidities such as dyslipidaemia and diabetes. Cases of hypertension were validated through the identification of an anti-hypertensive prescription, which correlated well with self-reporting. We were able to perform a number of sensitivity analyses, such as assessing an alternate DII score, assessing pro- vs. anti-inflammatory diet, and excluding cases temporally closer to the exposure, finding consistent associations.

As has been discussed, this study is observational thus it is difficult to rule-out residual confounding. Undiagnosed hypertension cases may have been missed, and could be a source of error, although we expect that these cases would attenuate any association as they would be randomly distributed. The DII was calculated from a detailed and validated dietary questionnaire, but the diet is subject to change over time, thus we are unable to account for dietary changes. As diet was self-reported, and is also associated with measurement error, it is possible that some participants were misclassified in terms of their DII. Importantly, we were unable to assess the effect of the DII on inflammatory markers, which is a major limitation; however the associations between the DII and inflammation have been well documented in the literature. The study included only women, and this may limit the generalizability of the study, however, we are not aware of any differences in metabolism that may mean that results would be different in men.

## Conclusion

In conclusion we observed weak positive associations between a highly pro-inflammatory diet, and the risk of hypertension, particularly in healthy-lean women. The foods most highly associated with a high DII were foods which have previously been implicated with adverse health outcomes, such as added sugars, fast food, and processed meats. These results support the idea that people at risk of hypertension and cardiovascular disease should be encouraged to make healthier food choices such as fruits and vegetables, and avoid consuming potentially inflammatory foods to reduce the pro-inflammatory potential of the diet.

## Supplementary information

**Additional file 1.**

**Additional file 2.**

## Data Availability

The datasets used and/or analysed during the current study are available from the corresponding author on reasonable request.
