# Supplementary material for: Dietary inflammatory index, risk of incident hypertension, and effect modification from BMI
Source: Nutr J. 2020 Jun 25;19:62. doi: 10.1186/s12937-020-00577-1 (PMC7315510; doi:10.1186/s12937-020-00577-1)
Supplement: Supplementary file 1 — Additional file 1. [file 12937_2020_577_MOESM1_ESM.docx]

**Supplement**

**Supplementary Table 1:** Dietary components included in the dietary inflammatory index

| Components | Dietary inflammatory index |
| --- | --- |
| Energy (kcal/d) | **˟** |
| Protein (g/d) | ✔ |
| Total fat (g/d) | **˟** |
| SFAs (g/d) | ✔ |
| MUFAs (g/d) | ✔ |
| PUFAs (g/d) | **˟** |
| Trans Fatty acids (g/d) | ✔ |
| n-3 PUFAs (g/d) | ✔ |
| n-6 PUFAs (g/d) | ✔ |
| Cholesterol (mg/d) | ✔ |
| Carbohydrate (g/d) | ✔ |
| Fiber (g/d) | ✔ |
| Ethanol (g/d) | ✔ |
| Caffeine (g/d) | ✔ |
| Vitamin A (µg/d) | ✔ |
| β-Carotene (µg/d) | ✔ |
| Thiamin (mg/d) | ✔ |
| Riboflavin (mg/d) | ✔ |
| Niacin (mg/d) | ✔ |
| Vitamin B-6 (µg/d) | ✔ |
| Folate (µg/d) | ✔ |
| Vitamin B-12 (µg/d) | ✔ |
| Vitamin C (mg/d) | ✔ |
| Vitamin D (µg/d) | ✔ |
| Vitamin E (mg/d) | ✔ |
| Iron (mg/d) | ✔ |
| Magnesium (mg/d) | ✔ |
| Zinc (mg/d) | ✔ |
| Tea (g/d) | ✔ |
| Eugenol (mg/d) | ✔ |
| Flavonols (mg/d) | ✔ |
| Flavonones (mg/d) | ✔ |
| Isoflavones (mg/d) | ✔ |
| Flavones (mg/d) | ✔ |
| Anthocyanidins (mg/d) | ✔ |

**Supplementary table 2**: BMI adjusted and multivariate adjusted cox proportional hazard models for incident hypertension risk based on distributions of adapted dietary inflammatory index standardised to the study population.

M0 with age as timescale; M1 adjusted for physical activity, smoking, family history of CVD, and education level; M2 M1 + diabetes and dyslipidaemia at baseline; M3 M2 + BMI

| **Quintiles of DII** | **Q1 (< -2.7)**  **(n = 9,331)** | **Q2 (-2.7 - -0.5)**  **(n = 9,330)** | **Q3 (-0.5 – 1.3)**  **(n = 9,330)** | **Q4 (1.3 – 3.0)**  **(n = 9,330)** | **Q5 (> 3.0)**  **(n = 9,331)** | **P for trend** |
| --- | --- | --- | --- | --- | --- | --- |
| Cases | 2,697 | 2,699 | 2,664 | 2,555 | 2,568 |  |
| Person years | 175,873 | 176,292 | 176,738 | 177,652 | 177,712 |  |
| *M0* | *ref* | 1.00 [0.95: 1.06] | 0.99 [0.94: 1.05] | 0.98 [0.92: 1.03] | 1.01 [0.96: 1.07] | 0.14 |
| *M1* | *ref* | 0.99 [0.94: 1.05] | 0.98 [0.93: 1.03] | 0.94 [0.89: 1.00] | 0.96 [0.91: 1.01] | **0.03** |
| *M2* | *ref* | 1.00 [0.95: 1.05] | 0.99 [0.94: 1.05] | 0.98 [0.92: 1.03] | 1.01 [0.95: 1.06] | 0.12 |
| *M3* | *ref* | 1.03 [0.97: 1.09] | 1.04 [0.99: 1.10] | 1.04 [0.99: 1.10] | 1.09 [1.03: 1.16] | **0.02** |

**Supplementary** **Table 3**: BMI adjusted and multivariate adjusted cox proportional hazard models for incident hypertension risk based on distributions of “non-adapted” dietary inflammatory index, according to the method of Shivappa (35).

M0 with age as timescale; M1 adjusted for physical activity, smoking, family history of CVD, total calories, and education level; M2 M1 + diabetes and dyslipidaemia at baseline; M3 M2 + BMI

| **Quintiles of DII** | **Q1 (< -2.7)**  **(n = 9,331)** | **Q2 (-2.7 - -0.4)**  **(n = 9,330)** | **Q3 (-0.4 – 1.3)**  **(n = 9,330)** | **Q4 (1.3 – 3.0)**  **(n = 9,330)** | **Q5 (> 3.0)**  **(n = 9,331)** | **P for trend** |
| --- | --- | --- | --- | --- | --- | --- |
| Cases | 2,706 | 2,697 | 2,583 | 2,568 | 2,565 |  |
| Person years | 173,855 | 175,138 | 176,262 | 175,993 | 175,773 |  |
| *M0* | *ref* | 0.99 [0.94: 1.04] | 0.94 [0.89: 1.00] | 0.94 [0.89: 0.99] | 0.94 [0.89: 0.99] | **0.002** |
| *M1* | *ref* | 0.99 [0.94: 1.04] | 0.94 [0.89: 1.00] | 0.94 [0.88: 1.00] | 0.94 [0.88: 1.00] | **0.02** |
| *M2* | *ref* | 1.00 [0.94: 1.05] | 0.95 [0.90: 1.01] | 0.95 [0.90: 1.01] | 0.96 [0.90: 1.02] | 0.07 |
| *M3* | *ref* | 1.04 [0.98: 1.09] | 1.02 [0.96: 1.08] | 1.04 [0.98: 1.10] | 1.07 [1.01: 1.14] | 0.06 |
